# Bilateral Anterior Ischemic Optic Neuropathy Accompanied with Unilateral Central Retinal Artery Occlusion in a Biopsy-proven Case of Giant Cell Arteritis

**DOI:** 10.18502/jovr.v17i3.11585

**Published:** 2022-08-15

**Authors:** Kaveh Abri Aghdam, Ali Aghajani, Mehdi Khakpour, Mostafa Soltan Sanjari

**Affiliations:** ^1^Eye Research Center, Eye Department, The Five Senses Health Institute, School of Medicine, Iran University of Medical Sciences, Tehran, Iran; ^2^Isfahan Eye Research center, Department of Ophthalmology, Isfahan University of Medical Sciences, Isfahan, Iran

##  PRESENTATION 

**Figure 1:**
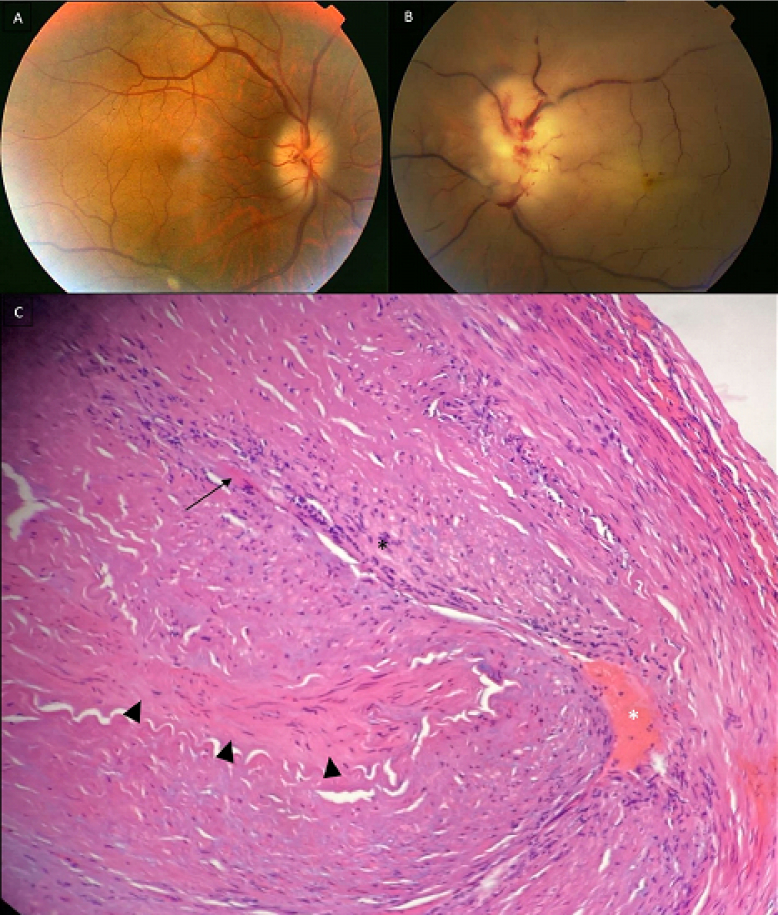
Color fundus photograph of the right eye showing a blurred-margin optic disc; note the paleness of the swollen optic disc (A). Color fundus photograph of the left eye showing diffuse opaque retina, severe arterial attenuation, box-carring, and chalky white blurred-margin optic disc with multiple superficial hemorrhages (B). Photomicrograph of the biopsy specimen from the left temporal artery reveals fibrinoid necrosis (arrowheads), endothelial destruction, and transmural infiltration of lymphocytes, polymorphonuclear neutrophils (black asterix), histiocytes, and giant cells (black arrow). Organized thrombi in the lumen are seen (white asterix). The overall findings are compatible with the diagnosis of giant cell arteritis (hematoxylin and eosin staining, 40X magnification) (C).

**Figure 2:**
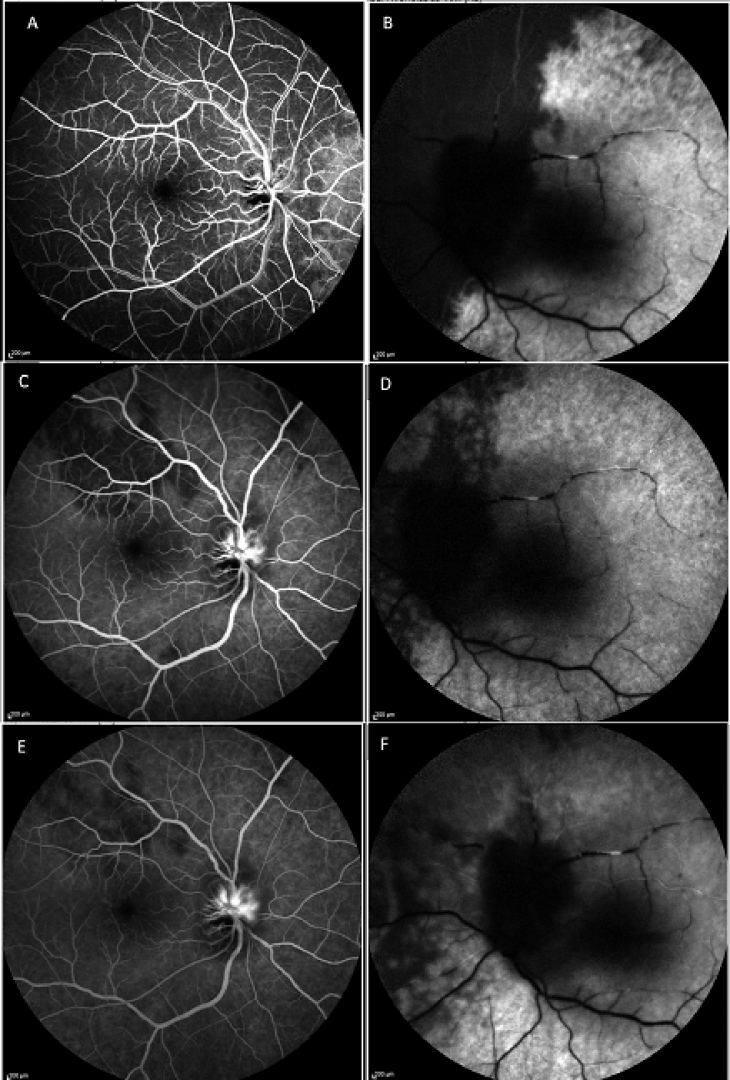
Fluorescein angiography in the early arteriovenous phase (30 sec) reveals a diffuse choroidal filling delay in the right eye (A), and a choroidal filling defect in the nasal retina as well as arterial filling delay in the left eye (B). Fluorescein angiography in the venous phase (1 min), patchy choroidal filling defects accompanied with optic disc leakage in the right eye (C), and a sectoral choroidal filling defect in the nasal fundus as well as arterial filling delay in the left eye (D). Fluorescein angiography in the late phase (5 min) shows patchy choroidal filling defect and optic nerve head leakage in the right eye (E), and patchy choroidal filling defect and arterial filling delay in the left eye (F).

An 80-year-old male patient was referred to the neuro-ophthalmology clinic with a history of sudden vision loss in the right eye one week earlier and vision loss in the left eye four days after that. The vision assessment was no light perception in both eyes at the time of the initial examination. Both eyes were pseudophakic and anterior segment examination was unremarkable. Funduscopic examination revealed a swollen optic disc in the right eye and a diffuse retinal whitening with a cherry-red spot, arterial attenuation, and a chalky-white blurred-margin optic disc in the left eye (Figures 1A & 1B). Fluorescein angiography (FA) disclosed a diffuse choroidal filling delay with leakage from the optic nerve head in the venous phase in the right eye and nasal choroidal filling delay and diffuse delay in the retinal arterial filling in the left eye [Figure 2]. The erythrocyte sedimentation rate was 98 mm/hr, and the level of C-reactive protein was 76 mg/L. The patient was admitted and pulse corticosteroid therapy was commenced with the diagnosis of giant cell arteritis (GCA). The diagnosis was later confirmed with a histopathologic evaluation of the left side temporal artery biopsy specimen [Figure 1C]. Unfortunately, even though treatment was started immediately, it failed to improve the patient's vision.

##  DISCUSSION 

Giant cell arteritis (GCA) is a granulomatous vasculitis that involves large- and medium-sized vessels. Anterior ischemic optic neuropathy (AION), central retinal artery occlusion (CRAO), and posterior ischemic optic neuropathy (PION) are the most reported ophthalmic presentations of GCA.^[1]^ The main goal of corticosteroid therapy in GCA is to prevent the occurrence or progression of visual loss. Bilateral ocular involvement is another concern in these patients. Hayreh et al^[2]^ reported that in all GCA patients with bilateral vision impairment, older changes could be found in one eye, suggesting that the patients were unaware of vision loss in one eye until the second eye was affected giving the erroneous interpretation of simultaneous bilateral ocular involvement. Thus, the estimations of the prevalence of simultaneous bilateral GCA ocular involvement suffer from the inaccuracy in the timing of the examination and perhaps were mostly imprecise and possibly preventable.

CRAO is a rare but well-known disaster that has been reported in 4% of GCA patients.^[3]^ CRAO in these patients is almost invariably associated with AION, and posterior ciliary artery occlusion (revealed by FA study in patients with CRAO) is highly suggestive of the arteritic nature of the occlusion.^[4]^ There has been only one report of a similar presentation of GCA in literature.^[5]^ Although we cannot confirm the similarity of the extent of bilateral involvement between these two cases due to the lack of sufficient angiographic data, both reports have one thing in common, which is neither of these patients has enjoyed vision improvement after pulse corticosteroid therapy. Overall, this report presents an extreme manifestation of GCA with detailed illustrations to emphasize the importance of timely diagnosis and urgent treatment in these patients.

##  Consent to Participate

Informed consent including publication of photographs in medical journals was obtained from the participant of this study.

##  Financial Support and Sponsorship

The authors did not receive any financial support or funding for this work.

##  Conflicts of Interest 

The authors have no conflicts of interest to declare.
